# *Ehrlichia*, *Hepatozoon*, and *Babesia* Coinfection Patterns Among Owned Dogs in Central Thailand

**DOI:** 10.1111/jvim.70154

**Published:** 2025-05-30

**Authors:** Rungrote Osathanon, Aeknarin Saechin, Chalisa Mongkolphan, Benjaporn Bhusri, Siriporn Tangsudjai

**Affiliations:** ^1^ Department of Veterinary Clinical Sciences and Public Health, Faculty of Veterinary Sciences Mahidol University Nakhon Pathom Thailand; ^2^ Monitoring and Surveillance Center for Zoonotic Diseases in Wildlife and Exotic Animals, Faculty of Veterinary Science Mahidol University Nakhon Pathom Thailand

**Keywords:** babesiosis, ehrlichiosis, hepatozoonosis, prevention of tick‐borne diseases, risk factors, tick‐borne coinfection

## Abstract

**Background:**

*Ehrlichia*, *Hepatozoon*, and *Babesia* have the potential to cause life‐threatening illnesses in dogs, especially when coinfections occur.

**Hypothesis/Objectives:**

To determine the infection rates, coinfection patterns, and risk factors associated with these pathogens in central Thailand.

**Animals:**

Two thousand five hundred nineteen owned dogs presented with clinical abnormalities consistent with tick‐borne diseases between 2019 and 2023.

**Methods:**

Retrospective study, blood samples were analyzed using multiplex PCR to assess infection rates. The study compared infection rates across different sexes and age groups and tracked monthly variations.

**Results:**

A total of 19.02% (95% CI: 17.50–20.60) of dogs were infected by one pathogen infection: *Ehrlichia* 11.47% (10.25–12.78), *Babesia* 2.78% (2.17–3.50), and *Hepatozoon* 1.83% (1.34–2.43). Infections occurred year‐round but peaked in May. Coinfections were observed in 2.94% (2.31–3.67) of cases. Among infected dogs, coinfections were identified in 34% (36/106) and 53% (52/98) of dogs with babesiosis or hepatozoonosis, respectively, whereas 19% (69/358) of dogs with ehrlichiosis were co‐infected. Coinfections peaked in April, occurred in all age groups, and were more frequent in dogs younger than 1 year compared to dogs older than 7 years.

**Conclusions and Clinical Importance:**

These findings underscore the importance of comprehensive diagnostic testing for multiple pathogens and tick prevention in dogs living in this region.

**Abbreviations**
B: *Babesia*
BLASTn: Basic Local Alignment Search Tool for nucleotide
dNTPs: deoxynucleotide triphosphates
E: *Ehrlichia*
H: *Hepatozoon*
MoZWE: Monitoring and Surveillance Center for Zoonotic Diseases in Wildlife and Exotic Animals

## 1 Introduction

The increasing popularity of pet ownership and prevalence of stray dogs promotes the transmission of tick‐borne diseases to both humans and pets in Southeast Asia [1]. *Ehrlichia* spp., *Hepatozoon* spp., and *Babesia* spp. are common pathogens in dogs. *Ehrlichia canis* is a globally recognized pathogen that affects dogs across continents [2, 3, 4]. *E. canis* is transmitted through the saliva of *Rhipicephalus sanguineus* ( *R. sanguineus* ) ticks. *E. canis* infects monocytes and macrophages and causes various clinical abnormalities including pyrexia, weight loss, lymphadenomegaly, anemia, and organomegaly [5, 6]. *Hepatozoon canis* and *Hepatozoon americanum* are transmitted by *R. sanguineus* and *Amblyomma maculatum* , respectively [7, 8]. Infection occurs by eating ticks carrying mature oocysts containing infective sporozoites. *H. canis* mostly infects hemolymphatic tissues, causing pyrexia, anemia, and lethargy [9, 10] whereas *H. americanum* targets muscular tissues, resulting in severe myositis and gait abnormalities [11]. Canine babesiosis is caused by intraerythrocytic protozoa of the genus *Babesia*. Large *Babesia* species that infect dogs include *B. canis* , *B*. *vogeli*, and *B. rossi* , whereas small *Babesia* species include *B*. *conradae, B*. *gibsoni*, and *B*. *vulpes*. These species exhibit distinct geographical distributions and varying pathogenicity [12]. The transmission of *Babesia* occurs through the bite of hard tick vectors (Ixodidae). The clinical manifestations vary in severity and are influenced by factors such as the species of *Babesia*, in addition to the age and immune status of the host [13]. Common clinical manifestations include pyrexia, anorexia, lethargy, lymphadenopathy, and splenomegaly [14]. However, some infected dogs exhibit no clinical signs [15].

Coinfection occurs when a host is infected with multiple pathogens, either through a single tick bite transmitting multiple pathogens simultaneously or through multiple tick bites from different vectors, each carrying a distinct pathogen. The likelihood of coinfection depends on environmental factors that support the presence and activity of specific tick species, influencing their ability to acquire and transmit pathogens. Regional variation in coinfection has been reported [16, 17]. A study in the United States revealed that dogs seropositive for *Anaplasma* were 1.40 times more likely to also test seropositive for *Babesia* [16]. A study in South India detected various coinfections of *B. gibsoni*, *B. vogeli*, *H. canis* , and *E. canis* in dogs [17]. Coinfection often complicates clinical and laboratory diagnoses [18]. This can worsen disease severity. However, various aspects and factors promoting the spread of tick‐borne pathogens and coinfections remain poorly understood and require further epidemiological studies. This study aims to investigate the infection rates, coinfection patterns, and risk factors associated with *Ehrlichia*, *Hepatozoon*, and *Babesia* tick‐borne pathogens among owned dogs in central Thailand.

## 2 Materials and Methods

### 2.1 Study Design and Subject Selection

This retrospective study analyzed data collected from 2019 to 2023 on 2519 dogs suspected of being infected with *Ehrlichia*, *Hepatozoon*, or *Babesia*. The cases were identified through a combination of molecular laboratory records from the Monitoring and Surveillance Center for Zoonotic Diseases in Wildlife and Exotic Animals (MoZWE) and medical records retrieved from the database of Prasu Arthorn Veterinary Teaching Hospital, Faculty of Veterinary Science, Mahidol University. All medical records associated with samples submitted for *Ehrlichia*, *Hepatozoon*, and *Babesia* screening during the study period were manually reviewed to identify relevant cases. Cases were selected based on clinical suspicion, with inclusion criteria requiring a combination of clinical signs. Dogs were included if they presented with at least two of the following clinical signs: pyrexia, anorexia, muscle pain, bleeding disorders, anemia, or thrombocytopenia, along with a history of tick exposure, and tested for the presence of *Ehrlichia*, *Hepatozoon*, and *Babesia* using PCR.

### 2.2 Exclusion Criteria

Dogs were excluded if they lacked clinical signs or a history of tick exposure. Additionally, dogs undergoing routine wellness checks without suspected tick‐borne diseases were excluded from the study.

### 2.3 Sample Collection and Data Analysis

Blood samples were collected in EDTA‐coated tubes and subsequently submitted to the MoZWE laboratory for analysis. DNA was extracted from the blood samples at the time of collection and stored at −20°C until PCR testing was performed in batches within 2 days. Data pertaining to the sampled dogs were obtained from MoZWE. Among the 2519 dogs included in the study, infection rates by sex were determined for 2461 individuals. Age‐specific infection rates were determined among 2308 dogs with reported birth dates. These individuals were categorized into five age groups: puppies (< 1 year), young adults (1–3 years), adults (4–6 years), old (7–10 years), and very old (> 10 years). The monthly infection rates were assessed based on the dates of sample collection. Ethical approval for this study was granted by the Faculty of Veterinary Science, Mahidol University.

### 2.4 DNA Extraction and Multiplex PCR System

DNA was extracted from 200 μL of peripheral blood using the Genomic DNA Mini Kit (Geneaid, Taipei, Taiwan). Samples were incubated in lysis buffer (0.1 M NaCl, 10 mM Tris‐Cl [pH 8.0], 5% SDS) with 10 μL of proteinase K (10 mg/mL) for 20 min at 56°C. DNA extraction was performed according to the manufacturer's protocol, and DNA was dissolved in 50 μL of 10 mM Tris–HCl (pH 8.0) containing 1 mM EDTA. Extracted DNA was stored at −20°C until use. For multiplex PCR amplification, three primer pairs were used: the virB9 protein gene of *Ehrlichia* [19], the 18S rRNA gene of *Hepatozoon* [19], and the 18S rRNA gene of *Babesia* [20] (Table S1). The primer sequences were sourced from previous studies [19, 20]. The multiplex PCR assay was optimized and validated for use in this study. This included testing against known positive controls to confirm specificity and sensitivity in canine samples (Table S1). PCR products were verified by Sanger sequencing. The PCR reaction mixture consisted of 25 μL of 1× Multiplex PCR Master Mix (QIAGEN, Germany), 1 μL of each primer at a concentration of 0.2 μM, 5 μL of template DNA, and nuclease‐free water to a total reaction volume of 50 μL. Amplifications were performed using a C1000 Touch Thermal Cycler (Bio‐Rad, Hercules, CA, USA) with the following conditions: initial denaturation at 95°C for 15 min; 35 cycles of denaturation at 94°C for 45 s, annealing at 61°C for 45 s, extension at 72°C for 1 min; and a final extension at 72°C for 10 min. PCR products were separated on 2.0% agarose gels stained with GelRed (Biotium, Fremont, CA, USA) and visualized using a UV transilluminator. The expected amplicon sizes were 380 (*Ehrlichia*), 737 (*Hepatozoon*), and 525 bp (*Babesia*). Positive and negative controls were included at all stages of the experimental workflow. Positive controls were derived from blood samples of clinically confirmed cases of *E. canis* , *H. canis* , and *B. vogeli*, which were validated through prior sequencing. Negative controls included two distinct types: (1) a blank control, in which nuclease‐free water was substituted for DNA to detect potential contamination, and (2) a biological negative control, comprising DNA extracted from a dog verified as negative for all three pathogens through diagnostic testing. All PCRs were performed in a laboratory with designated physical separation between DNA extraction, PCR setup, and post‐PCR analysis to prevent cross‐contamination.

### 2.5 PCR Amplification and DNA Sequencing

To further characterize the pathogens involved, new PCR tests were performed on 10% of the positive samples from each of the following coinfection patterns: *Ehrlichia* and *Hepatozoon* (four samples), *Ehrlichia* and *Babesia* (two samples), *Hepatozoon* and *Babesia* (two samples), and *Ehrlichia*, *Hepatozoon*, and *Babesia* (two samples). Each sample was individually retested using PCR, and the resulting amplicons were sequenced. The virB9 gene was targeted for *Ehrlichia*, while the 18S rRNA gene was used for *Hepatozoon* and *Babesia* [19, 20]. The PCR reaction mixture consisted of 1 μL of template DNA, 2.5 μL of 10× Mg^2+^free buffer, 1.5 mM Mg^2+^ solution, 1 mM dNTPs, 0.5 μM each primer (Table S1), and 2.5 units of *i‐Taq* DNA polymerase (iNtRON Biotechnology Inc., Korea). The cycling conditions included initial denaturation at 94°C for 2 min, followed by 30 cycles of denaturation at 94°C for 20 s, annealing at 61°C for 20 s, and extension at 72°C for 60 s. A final extension was performed at 72°C for 7 min. PCR products were gel‐purified and sequenced using the Sanger method at the U2Bio sequencing service (U2Bio Co. Ltd., Korea). The resulting sequences were analyzed using BLASTn to identify the genera of the pathogens. The sequences were compared to the corresponding nucleotide sequences of *Ehrlichia* spp., *Hepatozoon* spp., and *Babesia* spp. in GenBank. All sequences were deposited in the GenBank database, with accession numbers provided in Table S2.

### 2.6 Statistical Analyses

The infection rate was calculated on the basis of a combination of 5 years of data from 2019 to 2023. In all cases, the Kolmogorov–Smirnov test was applied to test for a normal distribution. The infection rate values were reported with a 95% confidence level via the Clopper–Pearson score interval. Fisher's exact test was used to compare infection rates between sexes. Pearson's *χ* ^2^ test was applied to compare infection rates across months and among age groups. Pairwise comparisons of differences between specific age groups were evaluated using Fisher's exact test. Logistic regression was performed to assess the association between specific months and overall and coinfection rates. The proportions (*P*) of single infections and coinfections with each pathogen were investigated. To achieve this, we employed the following formulas:

$$\begin{array}{c} P_{\text{single}}=\\ \frac{\text{Number of single infections}}{\text{Number of single infections}+\text{Number of coinfections}}\times 100 \end{array}$$

$$\begin{array}{c} P_{\text{coinfection}}=\\ \frac{\text{Number of coinfections}}{\text{Number of single infections}+\text{Number of coinfections}}\times 100 \end{array}$$

Significance was established at a *p* value of 0.05 or less (unless otherwise specified) with a 95% CI. All the statistical calculations were performed using SPSS 27 (IBM Corp., Armonk, NY, USA).

## 3 Results

**FIGURE 1:**
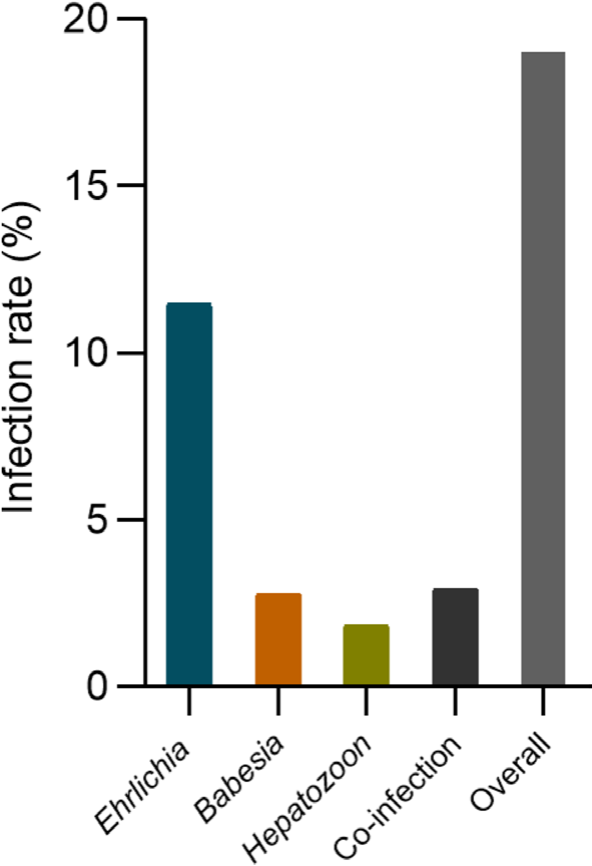
*Ehrlichia* (E), *Hepatozoon* (H), *Babesia* (B), coinfections, and the overall infection rate in dogs with suspected tick‐borne disease.

**FIGURE 2:**
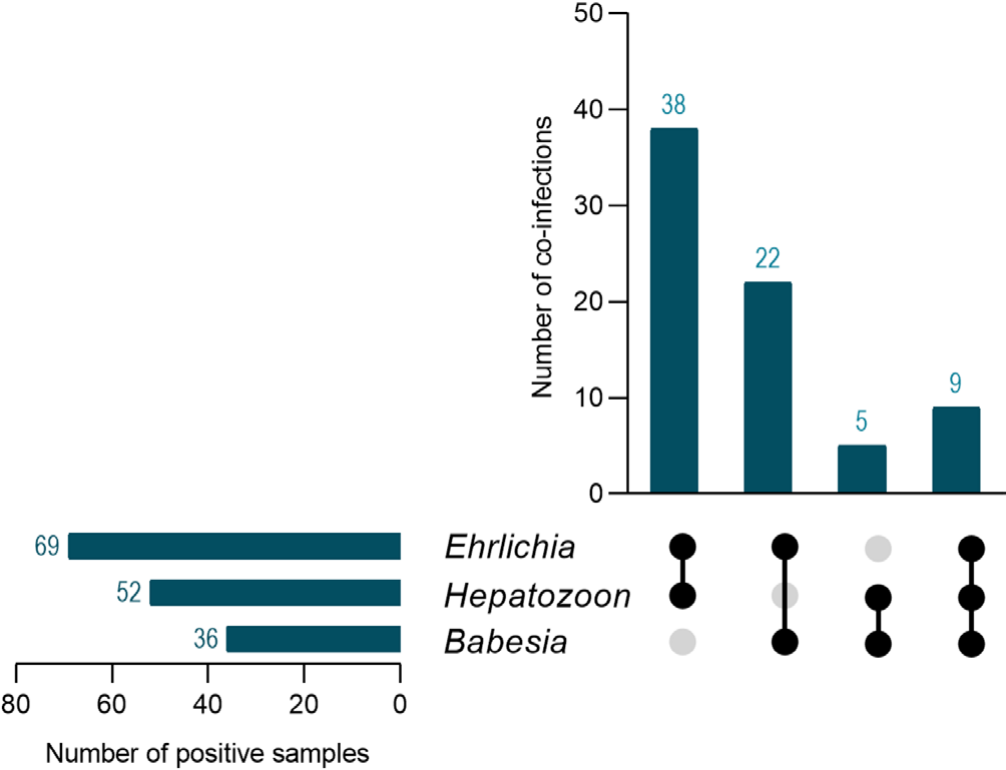
The numbers and patterns of *Ehrlichia*, *Hepatozoon*, and *Babesia* in co‐infected dogs. The horizontal bar graph represents the number of dogs infected with each individual pathogen. The vertical bar graph represents the number of dogs infected with each co‐pathogen.

**FIGURE 3:**
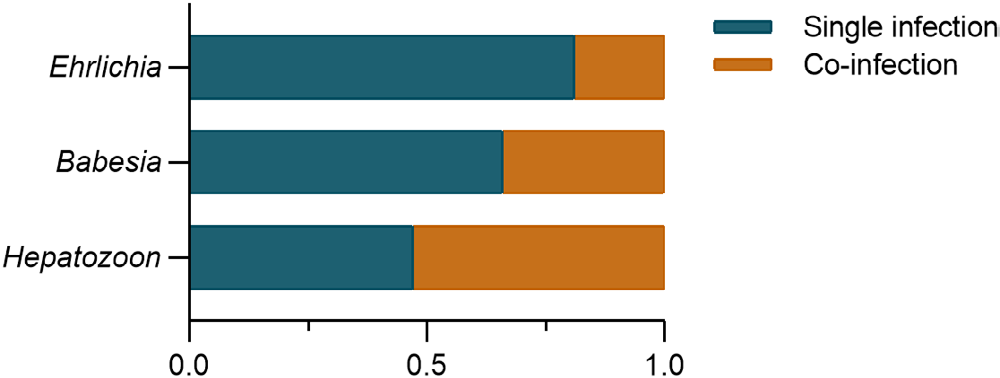
Proportions of single infections and coinfections with *Ehrlichia*, *Hepatozoon*, and *Babesia* in dogs. (based on infection rate data).

A retrospective analysis was conducted on 2519 dogs to assess the infection rates of *Ehrlichia*, *Hepatozoon*, and *Babesia* infections using multiplex PCR. Among these dogs, 479 (19.02%; 95% CI: 17.50–20.60) tested positive for at least one pathogen. *Ehrlichia* exhibited the highest infection rate with 289 cases (11.47%; 95% CI: 10.25–12.78), followed by *Babesia* with 70 cases (2.78%; 95% CI: 2.17–3.50), and *Hepatozoon* showed the lowest infection rate with 46 cases (1.83%; 95% CI: 1.34–2.43; Figure 1). Coinfections involving various combinations of *Ehrlichia*, *Hepatozoon*, and *Babesia* were observed with 74 cases (2.94%; 95% CI: 2.31–3.67). Coinfections between *Ehrlichia* and *Hepatozoon*, *Ehrlichia* and *Babesia*, and *Hepatozoon* and *Babesia*, as well as coinfections involving all three pathogens, were identified in this study (Figure 2; Table 1). The single and coinfection proportions of these three pathogens were analyzed. For *Babesia* and *Hepatozoon*, 34% (36/106) and 53% (52/98), respectively, were co‐infected. *Ehrlichia* was found in approximately 19% (69/358) of cases where there were coinfections with other pathogens (Figure 3).

**TABLE 1:** The number of infections with *Ehrlichia* (E), *Hepatozoon* (H), *Babesia* (B), and the number of coinfections (identification of 2–3 different genera of organisms in one sample) and the overall infection rate in 2519 dogs.

	E	H	B	E, H	E, B	H, B	E, H, B	Coinfection	Overall
Number of positive tests	289	46	70	38	22	5	9	74	479
Infection rate (%)	11.47	1.83	2.78	1.51	0.87	0.20	0.36	2.94	19.02

*Note:* The coinfection represents cases with infected pathogens from two or three different genera. Overall involving one pathogen and coinfection cases.

Ten percent of positive samples from each coinfection pattern were sequenced. BLASTn analysis revealed that all *Ehrlichia* coinfection sequences were 100% identical (227 bp) to the *E. canis* virB9 gene (GenBank AF546158.1, JF706287.1). The 18S rRNA sequences of *Hepatozoon* and *Babesia* coinfections were also 99.59%–100% identical (482 and 478 bp, respectively) to various *H. canis* (MK091086.1, EU289222.1) and *B. vogeli* (HM590440.1, DQ297390.1) genotypes in GenBank (Table S2).

There were also no statistically significant differences in individual and coinfection rates between males and females (Table S3). The overall infection rate was 19.54% (261/1336; 95% CI: 17.44–21.77) in male dogs and 18.76% (211/1125; 95% CI: 16.51–21.16) in female dogs, with no significant difference between the sexes (*p* = 0.62). *Ehrlichia* infection rates were the highest in both sexes, with males at 12.05% (95% CI: 10.35–13.92) and females at 10.93% (95% CI: 9.17–12.9). *Ehrlichia* had significantly higher infection rates than other detected organisms in both sexes (*p* < 0.001).

**FIGURE 4:**
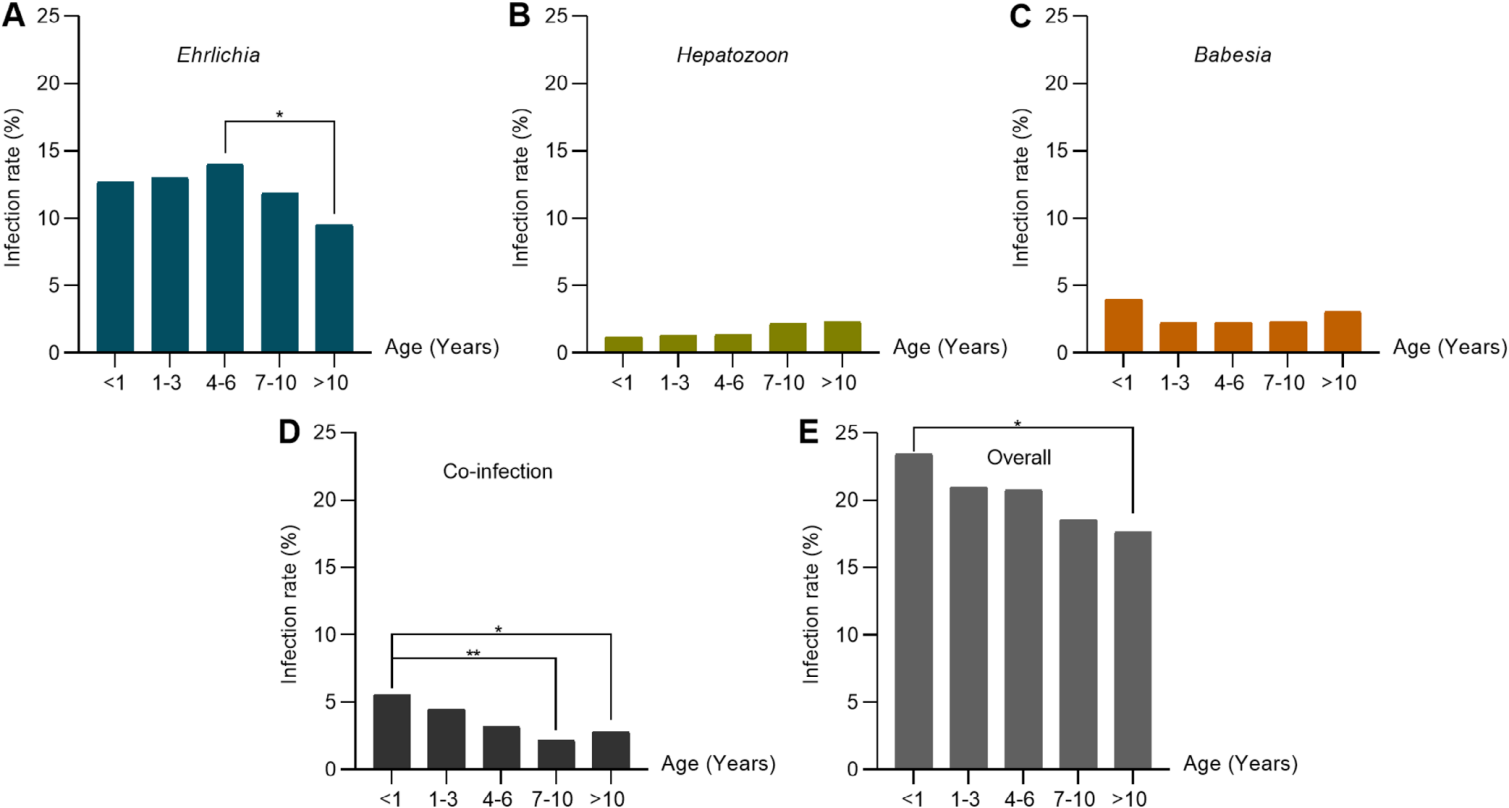
Bar charts illustrating the infection rates of tick‐borne pathogens across five different age groups for *Ehrlichia* (A), *Hepatozoon* (B), *Babesia* (C), coinfections (D), and overall infections (E). (*, **) indicates significant differences between groups (*p* ≤ 0.05, *p* < 0.01).

There were no significant differences in single‐pathogen or coinfection rates across age groups (Table S3). However, the overall infection rates differed between two age groups (*p* = 0.04): puppies (< 1 year) and very old dogs (> 10 years). Similarly, coinfection rates differed between dogs younger than 1 year and old‐aged dogs (7–10 years; *p* = 0.01), as well as between dogs younger than 1 year and very old dogs (> 10 years; *p* = 0.04; Figure 4).

**FIGURE 5:**
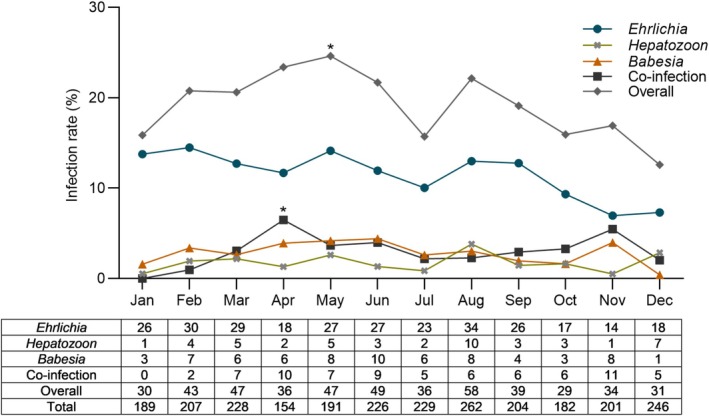
Average monthly infection rates in 2519 dogs presenting with clinical abnormalities consistent with tick‐borne disease between 2019 and 2023. The table indicates number of positive tests for each month. (*) indicates significant differences (*p* ≤ 0.05).

Infection rates of *Ehrlichia*, *Hepatozoon*, and *Babesia* did not differ across months (*p* = 0.18, *p* = 0.25, and *p* = 0.27, respectively). However, coinfection and overall infection rates varied (*p* = 0.02 and *p* = 0.04, respectively). To identify which specific months contributed to these variations, we conducted logistic regression analyses. This analysis indicated that April was significantly correlated with coinfection rates (*p* = 0.03), while May was significantly correlated with overall infection rates (*p* = 0.04; Figure 5).

## 4 Discussion

In this study, 19.02% of 2519 dogs with clinical findings suggestive of tick‐borne disease were infected with at least one agent. *Ehrlichia* was most common, being identified in 11.47% of dogs, followed by *Babesia* (2.78%) and *Hepatozoon* (1.83%). In addition to being the most commonly identified organism overall, *Ehrlichia* was also the most commonly identified organism among males and females, across all age groups and throughout the year. Coinfections were observed in 2.94% of cases. This coinfection rate is similar to the findings of Cloete and colleagues, who reported a comparable rate of coinfections involving *B. rossi* and *E. canis* (2.0%, 3/149) in dogs [21]. In this study, *Ehrlichia* and *Hepatozoon* (1.51%) were the most common combination. The difference in co‐infecting agents between studies may be attributed to geographic differences, as tick species and regional variations in pathogens could contribute to the observed variation in coinfection patterns. Additionally, our study investigated the proportions of *Ehrlichia*, *Hepatozoon*, and *Babesia*‐positive cases between single and coinfections. The study revealed that coinfection was common among dogs infected with *Babesia* and *Hepatozoon*, occurring in 34% (36/106) and 53% (52/98) of cases, respectively. These rates are higher than the 19% observed in *Ehrlichia*‐infected dogs. These findings illustrate the importance of comprehensive diagnostic testing for multiple tick‐borne pathogens in general and in individual patients, especially given that these pathogens require distinct therapeutic approaches.

Understanding relevant epidemiological factors, such as host demographics, environmental conditions, tick species distributions, and seasonal trends, is important in developing strategies and concentrating efforts to prevent and control the transmission of tick‐borne diseases. Our study suggested that sex factors did not increase the risk of *Ehrlichia*, *Hepatozoon*, *Babesia*, or coinfections. These findings are consistent with those of previous studies, which reported that *Ehrlichia*, *Hepatozoon*, and *Babesia* infections are widespread in both sexes [22, 23, 24]. However, some studies have noted a trend where *Ehrlichia* and *Babesia* infections may be more common in male dogs [22, 23], while *Hepatozoon* infections are often more prevalent in females [25]. These differences may vary due to different hormonal profiles, different environments, or dog behaviors that expose dogs to more ticks [24, 25]. Age also emerges as an important factor in the epidemiology of these infections. Our study revealed age‐related infection dynamics, with dogs younger than 1 year showing higher rates of coinfections compared to those older than 7 years, and higher overall infection rates compared to dogs older than 10 years. This suggests that age is an important factor to consider to understand and treat these infections in dogs. Future studies should explore the underlying mechanisms causing these patterns to reduce the burden of these infections across different age groups.

The study into the environmental and ecological drivers of tick‐borne pathogen transmission will help to predict disease prevalence and the prevalence of coinfection. Focusing on the monthly infection rates of tick‐borne pathogens *Ehrlichia*, *Hepatozoon*, *Babesia*, and coinfections reveals key insights into disease dynamics. Monthly variations in coinfection and overall infection rates indicate that complex interactions influence these patterns. Coinfection rates peak in April, during the seasonal transitions from summer to the rainy season. This pattern aligns with the period when the overall infection rate increases as summer transitions into the rainy season. Implementing targeted interventions, including comprehensive management of high‐risk populations, is essential, particularly before and during high‐risk periods. In combination with these strategies, year‐round preventive measures should be maintained to effectively reduce the tick‐borne disease prevalence. Future studies with larger sample sizes could offer more insights into the seasonal dynamics of these infections.

A limitation of this study is the inclusion of only dogs with a history of tick exposure and suspected cases, which may affect the generalizability of the findings. This selection criterion could underestimate the true prevalence of infections in the broader canine population. Future studies should adopt a more inclusive sampling approach to better capture the epidemiology of tick‐borne diseases across diverse populations. Additionally, the use of PCR testing alone poses limitations, as these organisms can circulate in blood at low numbers and intermittently, leading to potential false negatives. This could further contribute to an underestimation of the actual prevalence of infection. A negative PCR result does not rule out infection, and repeat testing, including serologic methods, may be necessary for accurate diagnosis [26, 27]. Furthermore, this study has limitations in understanding tick vector ecology, such as variations in tick species, geographic distribution, seasonal activity, and pathogen prevalence within vector populations. These factors may differ across regions and environmental conditions. Further research into vector dynamics and the environmental and ecological drivers of these patterns is essential to enhance our understanding and improve management strategies for tick‐borne diseases.

In conclusion, *Ehrlichia* was the most prevalent pathogen. There were coinfections involving two or three pathogens. *Ehrlichia* and *Hepatozoon* were the most common forms of coinfections. This study also found coinfections were more common in dogs younger than 1 year compared to dogs older than 7 years. Seasonal analysis revealed significant monthly variations in coinfection and overall infection rates, with April emerging as an important month for increased coinfections and May for higher overall infection rates during the transitional season. Implementing more effective prevention and control strategies, including year‐round tick prevention and comprehensive diagnostic testing, will be essential to reduce the burden of tick‐borne diseases in both dogs and potentially humans in the region.

## Disclosure

The authors declare no off‐label use of antimicrobials.

## Ethics Statement

Approved by the Faculty of Veterinary Science, Mahidol University‐Animal Care and Use Committee (MUVS‐ACUC), Thailand (approval number MUVS‐2024‐07‐43). Authors declare human ethics approval was not needed.

## Conflicts of Interest

The authors declare no conflicts of interest.

## Supporting information


**Table S1.** Supporting Information.

**Table S2.** Supporting Information.

**Table S3.** Supporting Information.
