# Supplementary material for: Ehrlichia, Hepatozoon, and Babesia Coinfection Patterns Among Owned Dogs in Central Thailand
Source: J Vet Intern Med. 2025 May 30;39(4):e70154. doi: 10.1111/jvim.70154 (PMC12124919; doi:10.1111/jvim.70154)
Supplement: Supplementary file 1 — Table S1. Supporting Information. [file JVIM-39-e70154-s002.docx]

**Supplementary Table 1** PCR primers and sequences

| Primer name | Primer sequence (5’-3’) | Gene | Ref |
| --- | --- | --- | --- |
| Ehr1401F | CCATAAGCATAGCTGATAACCCTGTTACAA | virB9 | Kledmanee, et al., 2009 |
| Ehr1780R | TGGATAATAAAACCGTACTATGTATGCTAG | virB9 | Kledmanee, et al., 2009 |
| Hep001F | CCTGGCTATACATGAGCAAAATCTCAACTT | 18S rRNA | Kledmanee, et al., 2009 |
| Hep737R | CCAACTGTCCCTATCAATCATTAAAGC | 18S rRNA | Kledmanee, et al., 2009 |
| Ba143-167 | CCGTGCTAATTGTAGGGCTAATACA | 18S rRNA | Spolidorio, et al., 2011 |
| Ba694-667 | GCTTGAAACACTCTARTTTCTCAAAG | 18S rRNA | Spolidorio, et al., 2011 |

The multiplex PCR (mPCR) assay was optimized and validated for use in this study. To determine the minimum detection limit, a mixture of three plasmids (pEhrl, pHep, and pBa) and each individual plasmid were serially diluted from 1 × 10⁶ to 1 × 10¹ copies/μl. Sensitivity testing showed that when detecting individual plasmids, the minimum detection limits were 1 × 10¹ copies for pEhrl and pBa, and 1 × 10² copies for pHep. When using the mixed plasmid template, the minimum detection limit for mPCR was 1 × 10² copies of each pathogen.

To evaluate the assay’s sensitivity and specificity, 60 confirmed DNA samples were tested. This set included 30 single-pathogen positive cases (10 each for *Ehrlichia*, *Hepatozoon*, and *Babesia*), 15 co-infection cases involving two-pathogen combinations (five for each pair), five cases of triple-pathogen co-infection, and five negative samples. The sensitivity and specificity for detecting a single pathogen were both 100%. For co-infections involving two or three pathogens, sensitivity was 95%, while specificity remained at 100%.
