# Supplementary material for: Ehrlichia, Hepatozoon, and Babesia Coinfection Patterns Among Owned Dogs in Central Thailand
Source: J Vet Intern Med. 2025 May 30;39(4):e70154. doi: 10.1111/jvim.70154 (PMC12124919; doi:10.1111/jvim.70154)
Supplement: Supplementary file 2 — Table S2. Supporting Information. [file JVIM-39-e70154-s003.docx]

| Sample Code | Highest Blastn Match | Reference Query | % Pairwise Similarlity | % Query Cover | Source | Country | Accession no. | Host |
| --- | --- | --- | --- | --- | --- | --- | --- | --- |
| 628E | Ehrlichia canis | JF706287.1 | 100 | 100 | Blood | Thailand | PQ153227 | Canis familiaris |
| 6002E | Ehrlichia canis | JF706287.1 | 100 | 100 | Blood | Thailand | PQ153228 | Canis familiaris |
| 2764E | Ehrlichia canis | JF706287.1 | 100 | 100 | Blood | Thailand | PQ153229 | Canis familiaris |
| 2443E | Ehrlichia canis | JF706287.1 | 100 | 100 | Blood | Thailand | PQ153230 | Canis familiaris |
| 6478E | Ehrlichia canis | JF706287.1 | 100 | 100 | Blood | Thailand | PQ153231 | Canis familiaris |
| 493E | Ehrlichia canis | JF706287.1 | 100 | 100 | Blood | Thailand | PQ153232 | Canis familiaris |
| 2255E | Ehrlichia canis | AF546158.1 | 100 | 100 | Blood | Thailand | PQ153233 | Canis familiaris |
| 4455E | Ehrlichia canis | AF546158.1 | 100 | 100 | Blood | Thailand | PQ153234 | Canis familiaris |
| 628H | Hepatozoon canis | MK091086.1 | 100 | 100 | Blood | Thailand | PQ157342 | Canis familiaris |
| 2764H | Hepatozoon canis | MK091086.1 | 100 | 100 | Blood | Thailand | PQ157343 | Canis familiaris |
| 493H | Hepatozoon canis | EU289222.1 | 99.79 | 100 | Blood | Thailand | PQ157344 | Canis familiaris |
| 4455H | Hepatozoon canis | MK091086.1 | 100 | 100 | Blood | Thailand | PQ157345 | Canis familiaris |
| 6075H | Hepatozoon canis | MK091086.1 | 99.79 | 100 | Blood | Thailand | PQ157346 | Canis familiaris |
| 4008H | Hepatozoon canis | MK091086.1 | 100 | 100 | Blood | Thailand | PQ157347 | Canis familiaris |
| 6478H | Hepatozoon canis | EU289222.1 | 99.59 | 100 | Blood | Thailand | PQ157348 | Canis familiaris |
| 2255H | Hepatozoon canis | EU289222.1 | 99.59 | 100 | Blood | Thailand | PQ157349 | Canis familiaris |
| 4424B | Babesia vogeli | HM590440.1 | 100 | 100 | Blood | Thailand | PQ157350 | Canis familiaris |
| 5227B | Babesia vogeli | HM590440.1 | 100 | 100 | Blood | Thailand | PQ157351 | Canis familiaris |
| 5569B | Babesia vogeli | DQ297390.1 | 100 | 100 | Blood | Thailand | PQ157352 | Canis familiaris |
| 644B | Babesia vogeli | DQ297390.1 | 100 | 100 | Blood | Thailand | PQ157353 | Canis familiaris |
| 735B | Babesia vogeli | HM590440.1 | 100 | 100 | Blood | Thailand | PQ157354 | Canis familiaris |
| 7497B | Babesia vogeli | HM590440.1 | 100 | 100 | Blood | Thailand | PQ157355 | Canis familiaris |

**Supplementary Table 2** Nucleotide sequence accession numbers
