# Supplementary material for: Ehrlichia, Hepatozoon, and Babesia Coinfection Patterns Among Owned Dogs in Central Thailand
Source: J Vet Intern Med. 2025 May 30;39(4):e70154. doi: 10.1111/jvim.70154 (PMC12124919; doi:10.1111/jvim.70154)
Supplement: Supplementary file 3 — Table S3. Supporting Information. [file JVIM-39-e70154-s001.docx]

**Supplementary Table 3** Infection rates for individual tick-borne pathogens, co-infections and overall infections by sex (A) and age group (B).

A.

|  | Male | | Female | | *p*-value |
| --- | --- | --- | --- | --- | --- |
|  | No. | % | No. | % |  |
| Ehrlichia | 161/1336 | 12.05 | 123/1125 | 10.93 | 0.39 |
| Hepatozoon | 20/1336 | 1.50 | 25/1125 | 2.22 | 0.18 |
| Babesia | 39/1336 | 2.92 | 30/1125 | 2.67 | 0.71 |
| Co-infection | 41/1336 | 3.07 | 33/1125 | 2.93 | 0.85 |
| Overall | 261/1336 | 19.54 | 211/1125 | 18.76 | 0.62 |

B.

|  | <1 year | | 1-3 year | | - 1. year | | 7-10 year | | *>*10 year | | *p*-value |
| --- | --- | --- | --- | --- | --- | --- | --- | --- | --- | --- | --- |
|  | No. | % | No. | % | No. | % | No. | % | No. | % |  |
| Ehrlichia | 32/252 | 12.70 | 29/224 | 12.95 | 62/443 | 14.00 | 87/733 | 11.87 | 62/652 | 9.51 | 0.21 |
| Hepatozoon | 3/252 | 1.19 | 3/224 | 1.34 | 6/443 | 1.35 | 16/733 | 2.18 | 15/652 | 2.30 | 0.61 |
| Babesia | 10/252 | 3.97 | 5/224 | 2.23 | 10/443 | 2.26 | 17/733 | 2.32 | 20/652 | 3.07 | 0.59 |
| Co-infection | 14/252 | 5.56 | 10/224 | 4.46 | 14/443 | 3.16 | 16/733 | 2.18 | 18/652 | 2.76 | 0.07 |
| Overall | 59/252 | 23.41 | 47/224 | 20.98 | 92/443 | 20.77 | 136/733 | 18.55 | 115/652 | 17.64 | 0.28 |

No. = Number of positive/Number of tested
